# TMJ Ankylosis in Children: A Case Report and Literature Review

**DOI:** 10.1155/2023/6474478

**Published:** 2023-01-05

**Authors:** Abdouldaim Ukwas, Mahmoud Elshik, Mohammed Elbialy

**Affiliations:** ^1^Division of Maxillofacial Diagnostic, Medical and Surgical Sciences, UCL Eastman Dental Institute, London, UK; ^2^Oral and Maxillofacial Surgery Department, Elhoria Specialized Hospital, El-Gharbya, Kafr el-Sheikh, Egypt; ^3^Oral and Maxillofacial Surgery Department, Kafr El-Eheikh General Hospital, Kafr El-Eheikh, Egypt

## Abstract

Temporomandibular joint (TMJ) ankylosis is a serious disabling condition characterized by fusion of the mandibular condyle with the glenoid fossa, disc, and/or eminence, resulting in severely restricted mouth opening and significantly reduced mandibular movements. The condition often has a deteriorating effect on the patient's daily functions such as speech, chewing, breathing, and oral hygiene as well as their wellbeing and quality of life. Furthermore, childhood TMJ ankylosis frequently has a detrimental impact on the facial growth resulting in facial asymmetry, micrognathia, and/or class II malocclusion with posterior or anterior open bite. Trauma is the main cause of TMJ ankylosis, but the condition can also occur as a result of surgery, local or systemic infections, or systemic disease. Surgery is the mainstay of treatment, and several approaches have been applied, including gap arthroplasty (GA), interpositional gap arthroplasty (IGA), reconstruction arthroplasty (RA), or distraction osteogenesis (DO). The aim of this article is to present a post-traumatic TMJ ankylosis case in a 5-year-old male child who was treated with resection and simultaneous costochondral grafting and to provide a succinct update of literature.

## 1. Introduction

The word “ankylosis” is of Greek origin (*ankylōsis* which means stiffening of joints). Temporomandibular joint (TMJ) ankylosis involves the fusion of the mandibular condyle and the glenoid fossa, disc, and/or eminence [1], which often results in a significantly reduced mobility of the mandible clinically manifesting by the inability to achieve a maximum incisal opening (MIO) greater than 5 mm as well as limited, if any, mandibular protrusion or lateral excursions, disabled mastication, and difficulty with breathing, speech, nutrition, and oral hygiene [2, 3]. Furthermore, childhood TMJ ankylosis often has a detrimental effect on the facial growth, resulting in facial asymmetry, mandibular micrognathia and bird-face deformity, and/or class II skeletal malocclusion with posterior or anterior open bite [4]. The condition is slightly more common in males than in females, and the ratio of unilateral to bilateral ankyloses is reported to be approximately 1.5 : 1 [5]. The most common cause of TMJ ankylosis is trauma, but the condition can also occur as a result of local and systemic infections, a systemic disease—such as ankylosing spondylitis, rheumatoid arthritis, and psoriasis—or a previous TMJ surgery [6]. Management of TMJ ankylosis is usually surgery and depends on the patient's age and any associated facial deformity. The commonly applied surgical approaches include gap arthroplasty (GA), interpositional gap arthroplasty (IGA), reconstruction arthroplasty (RA), and distraction osteogenesis (DO) ([7, 8]).

## 2. Case Report

A 5-year-old male child presented at the outpatient clinic complaining of limited mouth opening of about 2 years duration. History revealed that the condition had developed following a facial trauma at the age of 2 years when the child had fallen directly on his chin from a high position. Medical history of the patient was unremarkable.

**Figure 1:**
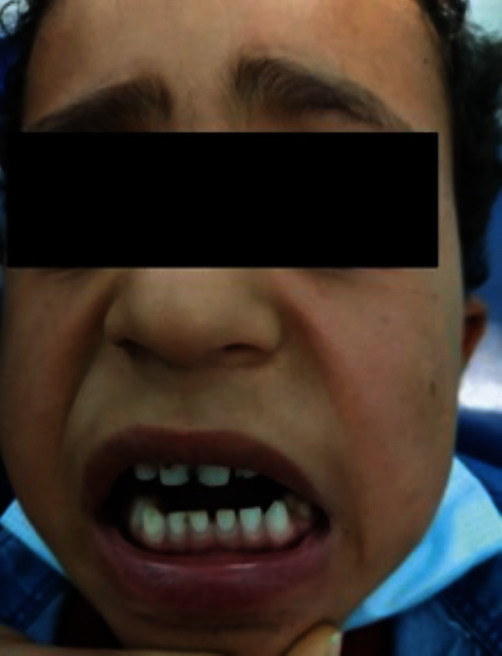
Front view showing facial asymmetry with mandibular deviation towards the left side.

Extraoral examination showed minimal facial asymmetry with slight mandibular deviation on opening towards the left side, no mandibular retrognathism, and hardly noticeable elongation of the face on the unaffected side (Figure 1). Examination of the TMJ revealed a significantly restricted mouth opening—inter-incisal mouth opening was 6 mm—significantly limited lateral excursive and protrusive movements, and deviation of the mandible towards the affected side upon opening. It was not possible to perform a proper intra-oral examination due to the extremely restricted mouth opening, but all of the child's deciduous teeth had erupted, and surprisingly only a few carious lesions were seen.

**Figure 2:**
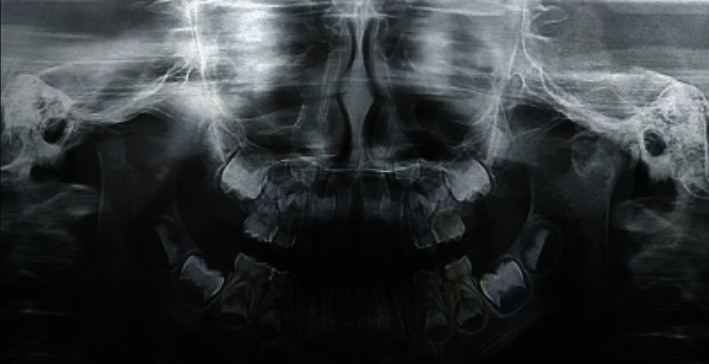
A pre-operative orthopantomography (OPG) of the patient.

**Figure 3:**
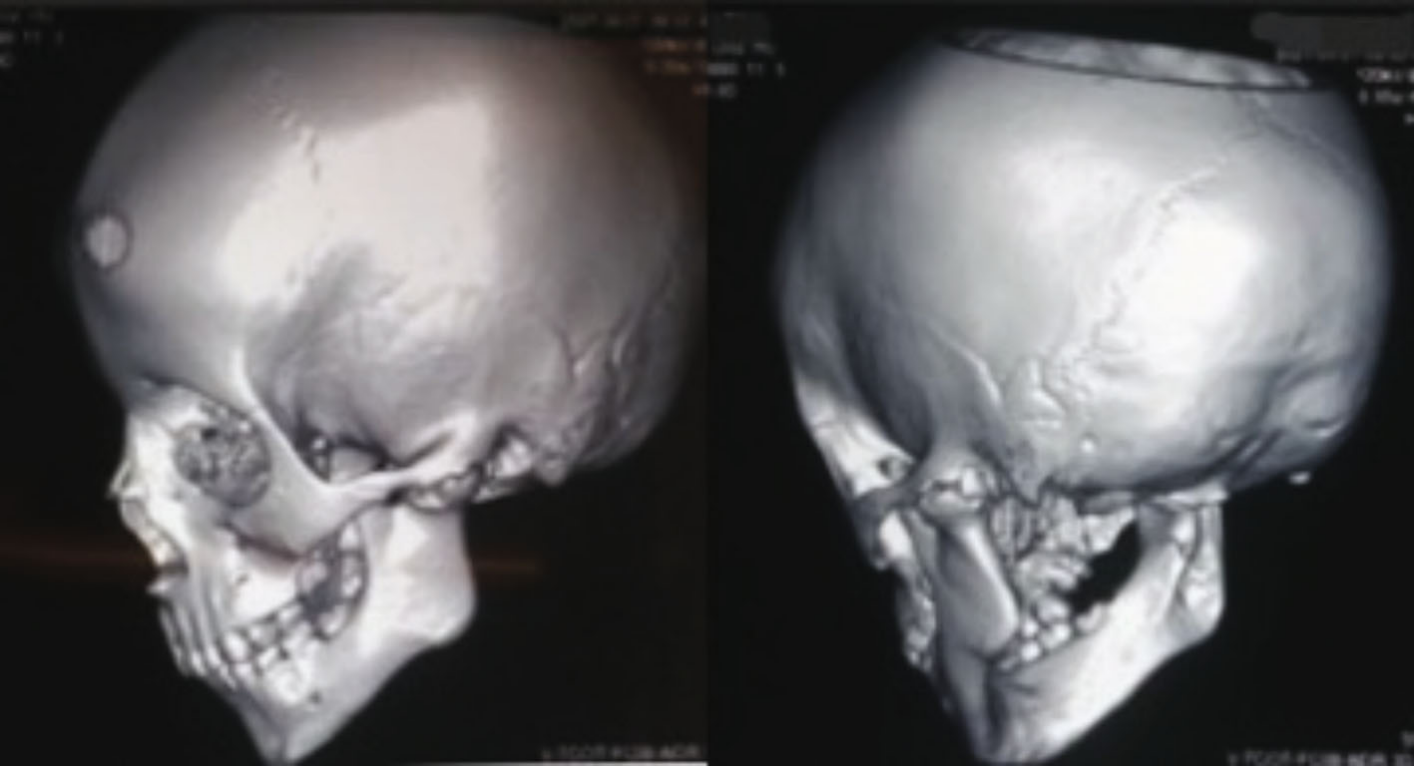
A 3-D reformatted CT scan of the patient showing left TMJ ankylosis and elongated left coronoid process.

**Figure 4:**
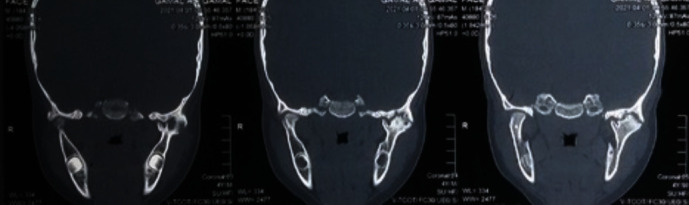
A coronal CT scan view showing left TMJ ankylosis.

An orthopantomagram (OPG) showed that the child had indeed all of his deciduous teeth, with evidence of several growing permanent teeth buds corresponding to the patient's age. A narrowed TMJ space and evidence of left TMJ ankylosis was detected (Figure 2), which was subsequently confirmed by coronal views and a 3-D reformatted computed tomography scans (Figures 3 and 4).

The child was eventually diagnosed with left TMJ ankylosis secondary to trauma, and a surgical intervention with resection and immediate reconstruction with costochondral rib graft (CCG) was decided.

**Figure 5:**
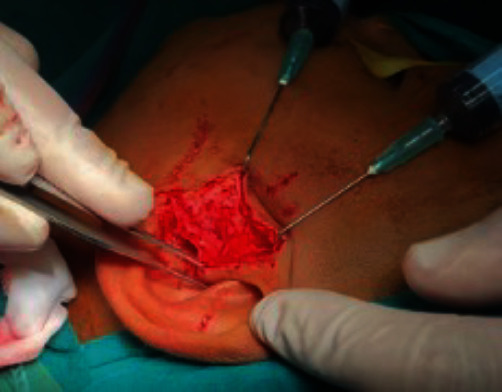
Endaural approach to access the ankylotic mass.

**Figure 6:**
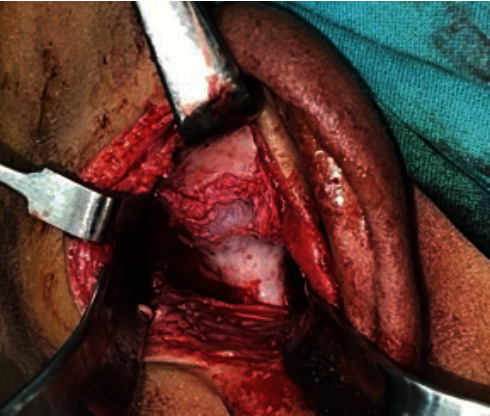
An intra-operative view of the ankylotic mass.

Due to the patient's extremely restricted mouth opening, the procedure was performed under general anaesthesia using fibre-optic-assisted nasotracheal intubation. After preparation of the surgical site, the ankylotic left TMJ was approached using endaural approach (Figure 5). The incision was deepened to the superficial temporalis fascia using a combination of blunt and sharp dissection. The flap was raised up to the zygomatic arch, and subsequently, the periosteum was incised on the most posterior aspect of the zygomatic arch. A subperiosteal plane of dissection was performed until the hard sclerotic mass was visible (Figure 6).

**Figure 7:**
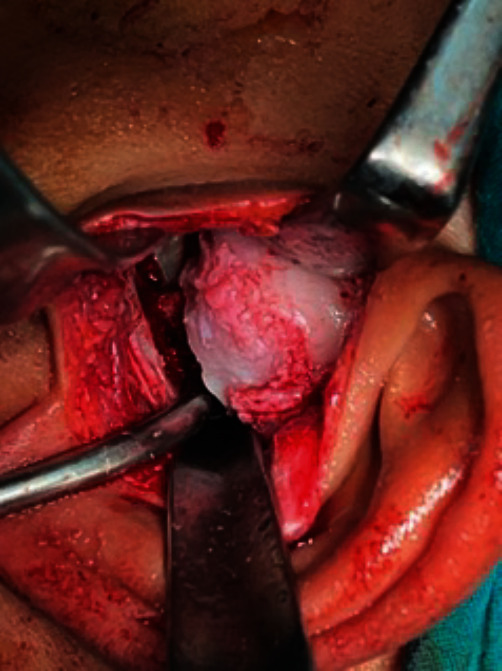
Osteotomy of the ankylotic mass.

A dense, hard sclerotic bony mass was discovered around the left TMJ. An osteotomy inferior to the ankylotic mass was done using a Lindemann bur while the tissues and vasculature medial to the ramus were protected using anterior and posterior Dunn-Dautrey retractors (Figure 7).

**Figure 8:**
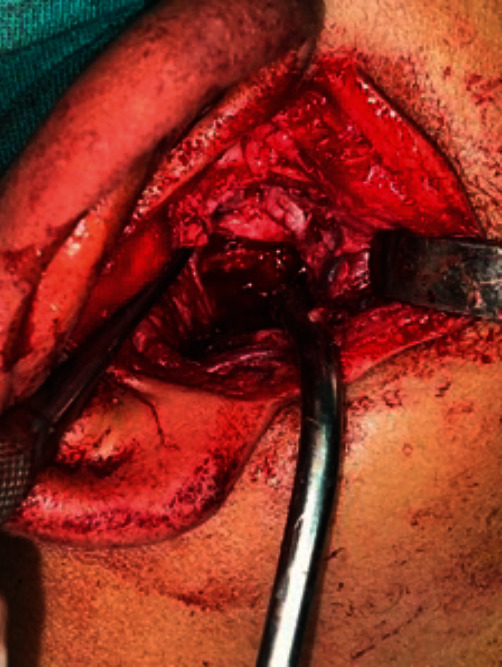
A gap is created following the removal of the ankylotic mass.

The ankylotic mass was subsequently removed, and bone was recontoured and smoothed creating a gap of about 1 cm between roof of glenoid fossa and cut end of the mandible (Figure 8).

**Figure 9:**
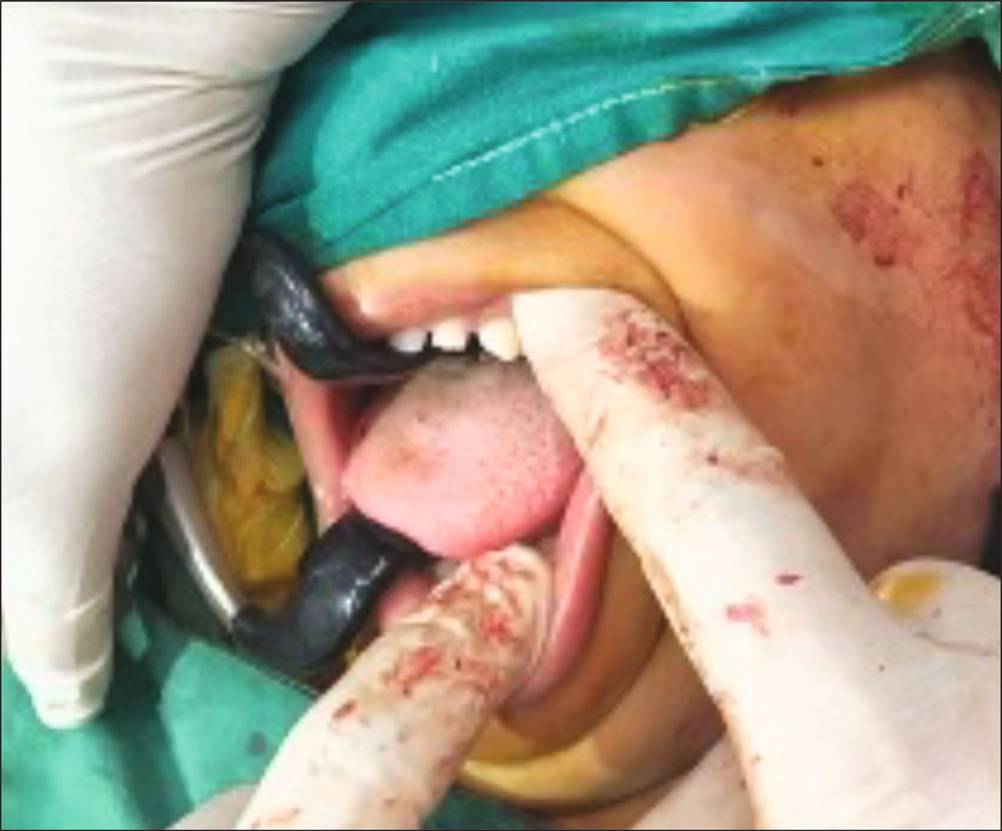
Inter-incisal opening following removal of the ankylotic mass.

The TMJ was then manipulated, and an inter-incisal distance of approximately 35 mm (the width of the surgeon's 3 fingers) was achieved (Figure 9). Decision was taken not to carry out coronoidectomy as the restricted mouth opening was sufficiently released.

**Figure 10:**
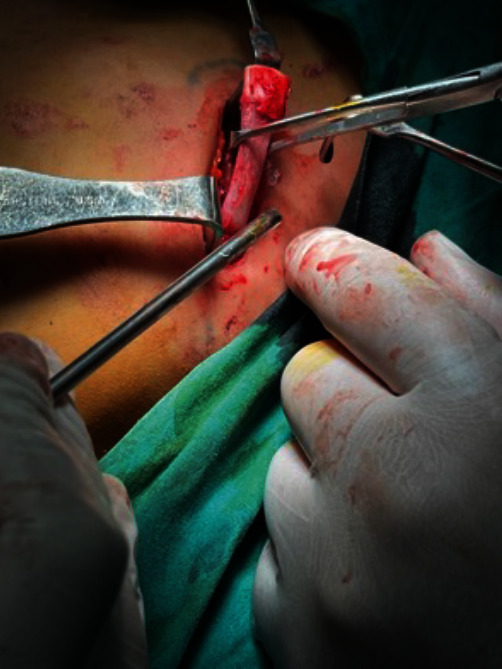
Harvesting the costochondral graft.

**Figure 11:**
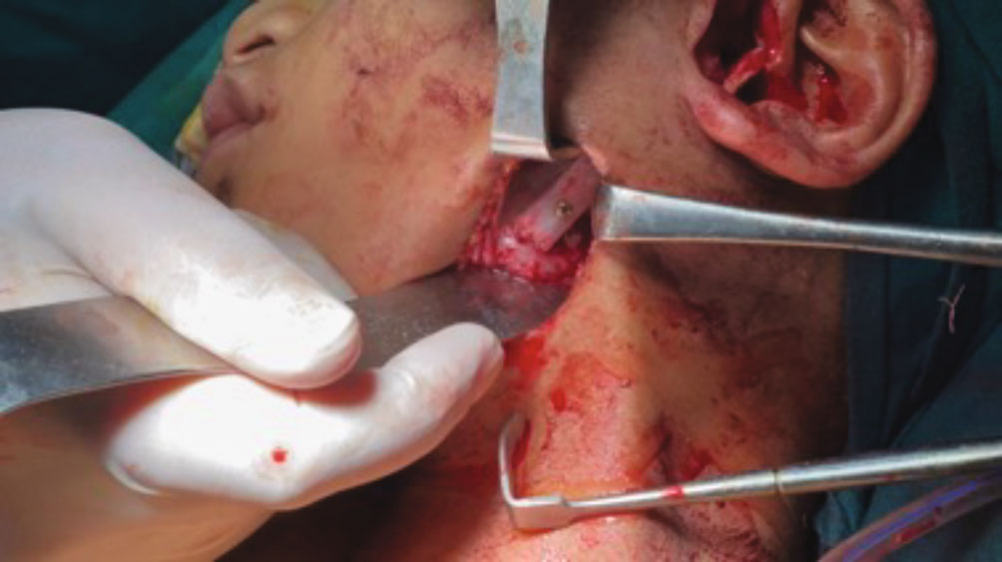
The graft is secured to the lateral side of the ramus.

Subsequently, a straight incision over the fifth rib was carried out, and around 8 mm length of the right fifth rib along with attached chondral part was harvested (Figure 10). The attached perichondrium was left in place without being stripped off to protect the cartilage from being sheared off the harvested rib upon functioning. The harvested graft was subsequently secured laterally to the left mandibular ramus through a submandibular incision, and the cartilage component was confirmed to be seated on the left glenoid fossa after slight smoothening of the edges of the costal cartilage. The graft was fixed with 3 bicortical screws in a line to the lateral side of the ramus without complete tightening of the middle screw to protect the curved from fracture from excessive force of the screw on the middle of its harvested length (Figure 11).

**Figure 12:**
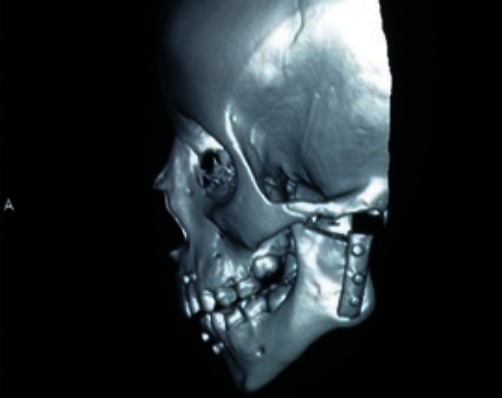
A post-operative 3-D reformatted image of a CT scan showing the graft in place.

Post-operatively, the patient's healing was uneventful except for noticeable paresis of the temporal branch of facial nerve that resolved over the following few weeks. The patient was discharged after 48 hours and was placed under strict physiotherapy exercise commenced 2 days post-operatively, and followed up for 6 months showing stable results. A CT scan taken 2 weeks post-operatively revealed that graft was stable in place (Figure 12).

## 3. Discussion

Temporomandibular joint ankylosis is a serious disabling condition that has a significant impact on the patient's quality of life, with deteriorating effects on nutrition, speech, oral hygiene, growth, occlusion, facial aesthetics, and—in severe cases—can cause micrognathia, which could lead to obstructive sleep apnoea. In children, unilateral ankylosis of TMJ often results in facial asymmetry due to deviation of the chin towards the affected side, which often has a compromised mandibular growth [9]. The condition can be classified according to the site (intra or extra-articular), the type of tissue involved (bony, fibrous, or fibro-osseous tissue), or the degree of fusion (complete or incomplete) [10]. TMJ ankylosis had also been classified into “true ankylosis”, where a condition (e.g., trauma, infection, or arthritis) promotes bony or fibrous adhesions within the TMJ capsule; or “false ankylosis” (also called pseudoankylosis) if joint movements are restricted by pathologies not related to the joint components, e.g., muscular or neurologic disorders [11].

The mandibular condyles in children are more prone to intra-capsular fractures due to trauma or surgical manipulation because they have a broad head and a relatively narrow neck [2]. Moreover, paediatric condyles have a high regenerative and remodeling capacity, which significantly increases the likelihood of ankylosis after trauma, or re-ankylosis after surgery [12].

Management of TMJ ankylosis is primarily surgical. Different surgical approaches have been described including GA, IGA, RA, and DO [8]. Surgery usually depends on the patient's age and the associated deformity [5, 13], and the choice of the surgical approach is often dictated by the clinician's preference and/or experience. There is no expert consensus or published guidelines for the management of TMJ ankylosis [1]. However, some authors recommend the use of GA for Sawhney types I and II, and interposition arthroplasty for types III and IV [3]. Furthermore, a 7-step protocol for management of TMJ ankylosis in children was published in 2009. The protocol includes aggressive resection of the fibrotic and/or bony mass, ipsilateral coronoidectomy, contralateral coronoidectomy—if inter-incisal opening was less than 35 mm after the resection and ipsilateral coronoidectomy—lining of the TMJ with temporalis fascia or disc (if it can be salvaged), reconstruction of the ramus condylar unit (RCU) with a CCG or DO and rigid fixation, early mobilization of the jaw, and aggressive physiotherapy [14]. However, no surgical approach has consistently achieved the optimum results.

Management of TMJ ankylosis in children is often challenging due to the unpredictability of the mandibular growth and the high rate of recurrence of ankylosis in children [3]. The challenge also extends to the anaesthesiologists who manage patient's airway [3]. The patient in our case was treated with resection and reconstruction with a CCG. This approach has many advantages including graft biocompatibility, functional adaptability, and CCG growth potential, which makes it an ideal choice for paediatric patients [5]. Several studies have shown that CCG can grow at a rate comparable to that of normal condyle due to the presence of primary and secondary growth centres, which indicates that the graft has intrinsic as well as adaptive growth potentials [15]. The main aim of grafting the resected condylar stump at this age is re-establishing posterior ramal height with the graft's growth potential. The graft itself does not affect movements, and the range of motion in this patient has substantially improved due to the release of the ankylosis. However, the mandible in this case deviates towards the left side (affected side) because of the lack of the left lateral pterygoid action, which leaves the mandible under the effect of the right lateral pterygoid muscle only. Furthermore, except for slight smoothening of the costal cartilage margins, no special treatment for the cartilage morphology was performed, and the perichondrium was left attached to the graft because the costal cartilage is usually fragile at this age. Current evidence does not support re-shaping of the costal cartilage, especially in young children, because it is assumed that it will undergo natural remodeling processes under forces while functioning [2, 14, 16].

But CCG has indeed some reported disadvantages such as fracture, donor site morbidity, increased operating time, unpredictable growth of the graft (normal growth, undergrowth, overgrowth, or no growth), and suboptimal range of motion post-operatively [17]. A relatively recent systematic review has reported that only 10 of 96 (10.5%) of CCG grafts showed unfavourable growth patterns: undergrowth in 1 graft, overgrowth in 7 grafts, lateral overgrowth in 1 graft, and no growth in 1 graft [16]. Moreover, evidence of resorption and re-ankylosis was reported in 21 (22%) and 8 (8%) of the cases, respectively.

In conclusion, early diagnosis and surgical intervention of TMJ ankylosis, particularity in growing children, is of paramount importance to avoid impairment of growth and the potential development of a unilateral mandibular retrusion, disabling malocclusion, and/or facial asymmetry [6], which often result in a negative impact on the mental health and wellbeing of the child. Although there are no standard surgical concepts in TMJ ankylosis surgery, a sufficient surgical exposure, adequate resection, appropriately selected graft, early and aggressive physiotherapy, and patient compliance are widely accepted as major factors positively influencing treatment success [5].

## Data Availability

Data supporting this research article are available from the corresponding author or first author on reasonable request.
